# Pimping: a tradition of gendered disempowerment

**DOI:** 10.1186/s12909-019-1761-1

**Published:** 2019-10-03

**Authors:** David R. Chen, Kelsey C. Priest

**Affiliations:** 1 University of Washington School of Medicine, Seattle, WA USA; 2 Oregon Health & Science University School of Medicine, Portland, OR USA

**Keywords:** Pimping, Medical education, Pedagogy, Gender, Sexual harassment

## Abstract

The use of *pimping* as a method of teaching is widespread in the clinical phase of medical education. In this paper we consider *pimping’s* colloquial meanings and discuss how it was introduced into the language of medical education. We posit that such language reflects persistent gendered hierarchies in medicine, and we evaluate *pimping’s* pedagogical value. Finally, we call for an end to the term and the practice, and for a renewed emphasis on pedagogy in medical education.

## Background

Awareness is growing regarding the numerous challenges associated with gender equity in medicine. Notably, the National Academies of Science, Engineering, & Medicine released a landmark report on sexual harassment in academia, concluding that gender violence is a rampant and largely unaddressed issue in medicine [1]. Meanwhile, *pimping* occurs on a daily basis in the clinical phases of medical education. Though *pimping* is used as a synonym for *quizzing*, it is unmistakably laden with gendered implications. Here we discuss the colloquial meanings of *pimping* and the etiology of the term in medicine. We then evaluate *pimping* in relation to language and pedagogy. We argue that both the term and the practice should end.

## Main text

Within medicine, *pimping* is understood as a tongue-in-cheek term; outside of medicine it connotes gendered and racialized poverty, violence, and suffering. The Merriam-Webster dictionary defines a *pimp* as “a criminal who is associated with, usually exerts control over, and lives off the earnings of one or more prostitutes” [2]. *Pimping* is a form of sex trafficking—a human rights, public health, and medical issue. Sex workers are at increased risk for violations ranging from homicide to unlawful arrest and detention [3]. How did *pimping* become detached from its colloquial meanings to find its place in the lexicon of medical education?

*Pimping* is incorrectly referenced as deriving from “*Pümpfrage*,” which translates to “pump questions” in German, owing to an article published by Brancati in 1989 [4]. In “The Art of Pimping,” Brancati fictitiously attributes *pimping’s* first usages to historical figures, among them William Harvey, a seventeenth century physician from London: “They know nothing of Natural Philosophy, these pin-heads. Drunkards, sloths, their bellies filled with Mead and Ale. O that I might see them pimped!” [5]. In addition to providing a mythological basis for *pimping*, Brancati sets forth prescriptions for how it should take place: “Pimp questions,” he explains, “should come in rapid succession and be essentially unanswerable” [5]. A follow up essay also titled “The Art of Pimping” by Detsky in 2009 further described how *pimping* cements the attending-resident-student hierarchy. In describing *pimping* etiquette for attending physicians, he states: “Respect educational order. Never ask a medical student to respond to a question after a resident has answered incorrectly” [6]. Although Brancati’s and Detsky’s pieces were written in jest, they tread on fraught territory. In effect, they legitimize the place of *pimping* in medical education, while disregarding its negative effects, such as those relating to language and pedagogy.

### Language

Language is “both a product and an engine of human culture” and “is one of the most common mechanisms by which gender is constructed and reinforced” [7]. Similarly, *pimping* should be understood as “a product and an engine of” patriarchy in medicine [8]. At a practical level, the use of the word *pimping* in medicine cues a gendered hierarchy. The *pimp* (attending, fellow, or resident) is a supervisor who evaluates the student. The *pimped* (medical student) must appease the attending, a performance necessary for career advancement. The double entendre that *pimping* in medicine carries is not subtle. *Pimping* occurs within a historically male and rigidly hierarchical system, and much of one-on-one clinical teaching takes place in a private setting. Indeed, sexual harassment is more common in medicine than in science or engineering [1].

Given the gendered nature of *pimping*, it is surprising that various articles exploring *pimping’s* relationship to medical student mistreatment do not acknowledge that women and those of non-binary gender identities may experience the practice differently from men [9–13]. The scotoma generated toward the gendered aspects of *pimping* is a sign of desensitization within the medical community: “I wonder if the authors, and the medical community at large, are aware of how startling and demeaning this practice sounds to those outside the medical profession,” a retired teacher from Napa Valley muses [14].

### Pedagogy

*Pimping* is often invoked as a form of the Socratic method. However, the relationship between the two methods is complicated—the only definitive commonality is the oral question and answer format [12, 15]. Wear and colleagues drew a distinction between “good” and “malignant” *pimping* [16]. Kost and Chen further identified that “malignant” *pimping* induces shame and humiliation [17]. Similarly, Stoddard and O’Dell asserted that the difference between the Socratic method and *pimping* “lies in the intent of the questioner” and the presence or absence of psychological safety [15]. Congruent with this picture, a qualitative study observed that “image management” was more important to students than the “optimization of their learning” when being *pimped* [18], and an estimated 43% of graduating medical students reported experiencing public embarrassment during their medical school training [19].

Brancati’s “essentially unanswerable” questions that “come in rapid succession” [5] are surely “malignant”; but is there still a difference between “good” *pimping* and the Socratic method? Applying a method of philosophical inquiry developed over 2400 years ago to modern medicine is no small task. Whereas Socrates was a philosopher concerned with “truth, justice, and virtue” whose “questioning … was intended to place an individual’s beliefs under scrutiny that would ultimately lead to their refutation” [12], medical education is focused on the propagation of existing scientific knowledge and clinical competency. Whereas Socrates was “motivated by his recognition of his lack of knowledge of ‘heaps of things’ and his desire to rectify that ignorance by examining the knowledge claims of others” [20], an attending physician already has the knowledge they seek to share with the student. And whereas the intention of the questions posed by Socrates was to stimulate speculative dialogue, *pimping* questions in medicine serve to reinforce the training hierarchy and evaluate whether the student knows a given fact [12]. Perhaps when we speak of the Socratic method we are referring to a concept that has evolved to apply to the current era. Perhaps we can declare that a benevolent, thoughtfully-crafted, open-ended question can dutifully be labeled “Socratic.” While such a question may technically be considered “good” *pimping*, the Socratic method would circle back and implore us to ask: Why should such a label be desired?

The term *pimping* and the practice of *pimping* should end. *Pimping* should be replaced with “a practice of questioning that considers purpose, Socratic principles, and adult learning theories...” [17]. Clinical education ought to be approached as a discipline in its own right, in which clinical faculty seek to achieve competency in pedagogical techniques. For example, the five-step “microskills” model is widely applicable [21], and thoughtful approaches to bedside teaching have been proposed [22]. At our institutions, the term *directed questioning* signifies a deep exploration of student knowledge with the purpose of locating gaps and boundaries, which we suggest as a replacement for *pimping*—both as concept and nomenclature.

There are challenges to this vision. A study conducted at Johns Hopkins University in the Department of Medicine found that 45% of attendings had a positive attitude toward *pimping*, whereas only 20% of attendings viewed *pimping* as effective in their own teaching practice [23]. Why do attendings resort to *pimping* as an educational method? As it has been put, “Teaching in medicine is not always financially rewarding, generally does not increase one’s publication record, and increases a workload that is already draining for house staff and attending physicians” [24]. We believe that the practice of *pimping* is widespread in part because it requires virtually no training or preparation, and can be rapidly performed. Asking already-overworked attending physicians and residents to acquire new pedagogical skills may seem unreasonable. Therefore, ending *pimping* will require a renewed dedication to pedagogy at all levels, including institutionally and nationally. We believe that teaching medical students can and should be regarded as a rewarding discipline, both personally and professionally. The honorific title, “doctor,” does mean, after all, “to teach” [25].

## Conclusion

We argue that *pimping* in medicine poses problems related to language and pedagogy. In particular, we highlight the gendered nature of the term and how the practice contributes to a hostile learning environment. We call for an end to both the term and the practice, as well as a renewed focus on pedagogy in medical education. These efforts are small steps on a bigger, broader, and more challenging journey to address gender violence within medicine [26]. This voyage begins with critical reflection and an honest assessment of how gender violence is perpetuated. This includes recognizing the subtexts of the words we use and the effects of the ways we teach [7].

## Data Availability

Not applicable.
